# A process analysis of the CA3 subregion of the hippocampus

**DOI:** 10.3389/fncel.2013.00078

**Published:** 2013-05-27

**Authors:** Raymond P. Kesner

**Affiliations:** Department of Psychology, University of UtahSalt Lake City, UT, USA

**Keywords:** CA3, memory, pattern completion, rapid encoding, associative processes

## Abstract

From a behavioral perspective, the CA3a,b subregion of the hippocampus plays an important role in the encoding of new spatial information within short-term memory with a duration of seconds and minutes. This can easily be observed in tasks that require rapid encoding, novelty detection, one-trial short-term or working memory, and one-trial cued recall primarily for spatial information. These are tasks that have been assumed to reflect the operations of episodic memory and require interactions between CA3a,b and the dentate gyrus (DG) via mossy fiber inputs into the CA3a,b. The CA3a,b is also important for encoding of spatial information requiring the acquisition of arbitrary and relational associations. All these tasks are assumed to operate within an autoassociative network function of the CA3 region. The CA3a,b also supports retrieval of short-term memory information based on a spatial pattern completion process. Based on afferent inputs into CA3a,b from the DG via mossy fibers and afferents from the entorhinal cortex into CA3a,b as well as reciprocal connections with the septum, CA3a,b can bias the process of encoding utilizing the operation of spatial pattern separation and the process of retrieval utilizing the operation of pattern completion. The CA3a,b also supports sequential processing of information in cooperation with CA1 based on the Schaffer collateral output from CA3a,b to CA1. The CA3c function is in part based on modulation of the DG in supporting pattern separation processes.

## INTRODUCTION

This review article emphasizes the importance of behavioral functions of the CA3 subregion of the hippocampus. After an anatomical description of the CA3a,b,c network, the influence of an autoassociative network function of the CA3 region in supporting mnemonic functions is presented. In general terms, the data suggest that CA3a,b mediates the acquisition and encoding of spatial information within short-term memory with a duration of seconds and minutes. In the context of short-term memory, the CA3a,b mediates rapid encoding of especially spatial information, novelty detection, and one-trial cued recall (all forms of episodic memory). Also, CA3a,b supports the acquisition of paired-associate associations as well as arbitrary associations. It should be noted that CA3a,b can also be involved in short-term memory retrieval as evidenced by support for a pattern completion process. Finally, CA3c function is in part based on modulation of the dentate gyrus (DG) in supporting pattern separation processes. Spatial information represents the critical attribute or domain that is processed in CA3. I will emphasize that the mnemonic functions of the autoassociative network of CA3 depend upon inputs into CA3 from other brain regions within the hippocampus (i.e., DG) and brain regions outside the hippocampus (i.e., medial and lateral entorhinal cortex) as well as outputs from CA3 to brain regions within the hippocampus (i.e., DG, CA1) and brain regions outside the hippocampus (i.e., medial and lateral septum). **Table 1** summarizes the functions of the CA3 region.

**Table 1:** Functions of the CA3 subregion of the hippocampus.

Function	CA3a	CA3b	CA3c
Rapid encoding of novel information	X	X	
Paired-associate learning	X	X	
Cued recall and arbitrary associations	X	X	
Pattern completion and retrieval	X	X	
Contribution to temporal processing via modulation of CA1	X	X	
Biasing information processing based on an interaction between pattern completion derived from the perforant path input into CA3 and pattern separation derived from the mossy fiber input into CA3	X	X	
Modulation of CA1 in temporal processing	X	X	
Modulation of CA1 in consolidation	X	X	
Spatial pattern separation in conjunction with the dentate gyrus			X

## ANATOMY

**FIGURE 1:**
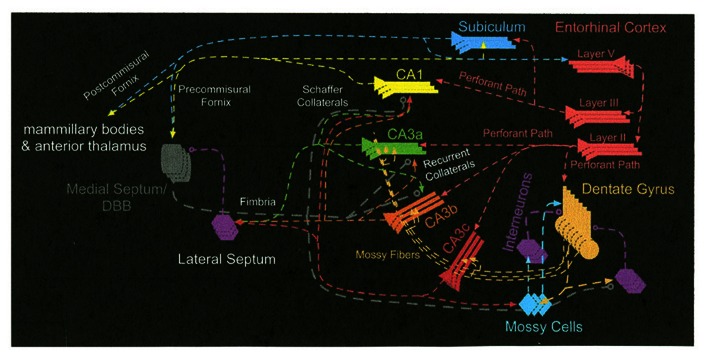
**The neural circuit that affects the operation of the CA3 subregion of the hippocampus**.

The most prominent anatomical feature of the CA3 subregion is that there are extensive interconnections among the principal cells via a recurrent collateral fiber system (Amaral and Witter, 1995). CA3 also receives converging inputs from multiple input pathways; for example, perforant path inputs from the medial and lateral entorhinal cortex, mossy fiber inputs from the DG, and its own outputs fed back as inputs via the recurrent collaterals (Amaral and Witter, 1995). In addition to the projections originating in CA1, projections out of Ammon’s horn originate in CA3. Many researchers have reported that CA3 projects to the lateral and medial septal nuclei as well as to the vertical limb of the diagonal band of Broca (Gaykema et al., 1991; Amaral and Witter, 1995; Risold and Swanson, 1997). The medial septum and vertical limb of the diagonal band of Broca, in turn, provides cholinergic and GABAergic inputs into the hippocampus (Amaral and Witter, 1995). It has been shown that the CA3 region can be divided into a CA3a, b, and c subareas (Lorente de Nó, 1934; Li et al., 1994). Based on the research of Li et al. (1994) and Buckmaster and Schwartzkroin (1994), it has been proposed that mossy cells receive excitatory inputs from granule cells and CA3c pyramidal cells and integrate the inputs from granule cells and CA3c pyramidal cells, which, in turn, via excitatory recurrent axonal projections activate many distal granule cells. Thus, CA3c may have a back-projection output that can influence the DG granule cells (Scharfman, 2007; Myers and Scharfman, 2011). It should also be noted that the CA3c regions do not have recurrent collaterals and it has a direct projection to CA1. Most of the synaptic connections embedded in those different pathways in CA3 are modifiable in their strength (Harris and Cotman, 1986; Marr, 1971; Zalutsky and Nicoll, 1990; Treves and Rolls, 1994; Debanne et al., 1998; Do et al., 2002; Nakazawa et al., 2002). These anatomical and physiological characteristics inspired many investigators to develop theoretical models to assign specific cognitive processes to CA3 (Marr, 1971; O’Reilly and McClelland, 1994; Treves and Rolls, 1994; Hasselmo et al., 1995, 2002a; Rolls, 1996; Hasselmo and Wyble, 1997; Samsonovich and McNaughton, 1997; Lisman, 1999; Mizumori et al., 1999; Kesner et al., 2004; Rolls and Kesner, 2006). The neural circuit that affects the operation of the CA3 subregion of the hippocampus is shown in **Figure 1**. 

## AUTOASSOCIATIVE NETWORK FUNCTIONS OF THE CA3 REGION

### RAPID ENCODING OF NOVEL INFORMATION

One of the mnemonic processes suggested for CA3a,b within short-term memory is the rapid acquisition of novel information. Marr proposed that the hippocampus should be capable of a rapid formation of simple representations, based on modifiable synaptic recurrent collateral associative connections among its neurons. This idea was further developed by McNaughton and Morris (1987) and Rolls (1989b) who have suggested that, based on CA3 recurrent collateral associative connections, the CA3 system may operate as an attractor network which is useful for some types of working memory. Support for this idea comes from a variety of studies analyzed by Kesner and Rolls (2001) and Rolls and Kesner (2006), including a study by Lee and Kesner (2002), who manipulated the *N*-methyl-D-aspartate (NMDA) receptors in CA3a,b by injecting dl-2-amino-5-phosphonovaleric acid (AP5), a pharmacological blocker for the NMDA receptors, selectively into CA3a,b in a delayed non-matching-to-place (DNMP) task. During a study phase of the task, rats visited a randomly chosen arm in the eight-arm maze and came back to the center platform where a delay period (10 s) was imposed in a bucket. After the delay period, the rats faced two adjacent arms on the maze (including the arm that had been visited before the delay period) and the task was to choose the arm that had not been visited during the previous study phase. Rats injected with AP5 were not impaired in performing the DNMP task in the familiar environment in which they had been trained. However, they were initially impaired in performing the task normally when the same task was carried out in a novel environment (i.e., on a novel maze in a novel testing room). AP5 injected into adjacent hippocampal subregions (e.g., DG or CA1) did not produce such deficits in the novel environment. In the same task CA3a,b, but not CA1, lesions disrupt performance with 10 s delays (Lee and Kesner, 2003). Similar results were obtained with CA3-specific neurotoxic lesions (Lee and Kesner, 2003). Nakazawa et al. (2003) also reported similar findings with a mouse strain in which the function of CA3 NMDA receptors was disrupted. These mutant mice were impaired in learning a novel platform location in a modified water maze task, whereas they were normal in finding familiar platform locations. In sum, these results strongly suggest that rapid, plastic changes in the CA3 network are essential for encoding novel information quickly into the hippocampal memory system and NMDA receptor-mediated plasticity mechanisms appear to play a significant role in the process.

Additional experimental evidence supporting the role of CA3a,b in rapid acquisition of novel information comes from a contextual fear-conditioning experiment with subregion-specific lesions (Lee and Kesner, 2004a). In this experiment, rats with CA3a,b-specific neurotoxic lesions were placed in a contextual fear-conditioning chamber (placed in a room with multiple visual landmarks and distinctive odors as contextual cues). The animals were given a 10-s tone stimulus footshock which co-terminated with a 2-s footshock. When the freezing response was measured during the intertrial interval period during acquisition (when only contextual cues were available), normal animals exhibited freezing behavior from the beginning. However, rats with CA3a,b lesions displayed a delayed onset of freezing behavior, suggesting that rapid formation of memory for the novel contextual cues was disrupted in these animals. Comparable results were reported by Cravens et al. (2006). They showed that rats with CA3 deletion of NMDA receptors failed to acquire a one-trial context discrimination task when tested 3 h, but not 24 h after acquisition.

Using a paradigm developed by Poucet (1989), rats with direct infusions of AP5 (an NMDA antagonist) or naloxone (a μ opiate antagonist) into the CA3a,b, were tested for the detection of a novel spatial configuration of familiar objects and the detection of a novel visual object change The results indicated that naloxone or AP5 infusions into the CA3a,b disrupted both novelty detection of a spatial location and a visual object (Hunsaker et al., 2007c).

Rapid encoding for novel object–place associations has also been shown to be mediated by CA3a,b in that lesions of CA3a,b totally disrupt performance of object-cued recall or place-cued recall tasks (Kesner et al., 2008). More detail is provided in the Section “Cued Recall and Arbitrary Associations.”

Finally, the CA3 network also seems essential in supporting the retrieval of information from memory when a short-term delay (e.g., 10 s) is introduced (Kesner and Rolls, 2001; Rolls and Kesner, 2006; Kesner et al., 2008). As the information circulates through the recurrent network in CA3, buffering of information within the network is likely to occur. Due to such holding phenomenon for neural activities within the recurrent network, CA3 may play a key role in short-term memory tasks especially when the nature of the tasks entails encoding of novel information or pattern completion. Most experimental results previously described here support this short-term memory function of CA3, since a short-term delay period was imposed in those tasks before the animals made choices. When the delay is prolonged exceeding the short-term range (e.g., 5 min or 24 h), CA3a,b did not remain as a key player in retrieving memory, because after the increased delay CA1 became more important (Lee and Kesner, 2003).

Recently, Lee et al. (2004) showed physiologically that the plasticity mechanism in CA3a,b was activated only when animals encountered novel configurations of familiar cues for the first time. Specifically, rats were trained to circle clockwise on a ring track whose surface was composed of four different textural cues (local cues). The ring track was positioned in the center of a curtained area in which various visual landmarks were also available along the curtained walls. To produce a novel cue configuration in the environment, distal landmarks and local cues on the track were rotated in opposite directions (distal landmarks were rotated clockwise and local cues were rotated counterclockwise by equal amounts). It is well known that principal cells in the hippocampus fire when the animal occupies a certain location of space, known as the “place field” of the cell. Mehta et al. (1997) originally showed that the location of the CA1 place field (measured by the center of mass of the place field) changed over time (shifting backward opposite to the direction of rat’s motion) in a familiar environment as the animal experienced the environment repeatedly. Such a prominent shift in the place field location appears to be associated with the NMDA receptor-dependent plasticity mechanism in the hippocampus (Ekstrom et al., 2001). When the rats encountered the changed cue configurations for the first time in the Lee et al. (2004) experiment, the CA3a,b place fields shifted their locations backward prominently compared to the place fields in CA1. However, such prominent shift was not observed in CA3a,b from day 2 onward (CA1 place fields started to exhibit a similar property from day 2). This double dissociation in the time course of plasticity between CA1 and CA3a,b place fields suggests that CA3a,b reacts rapidly to any changed components in the environment, presumably to incorporate the novel components into an existing system (or build a new representation of the environment if changes are significant).

Finally, single unit activity has been recorded in CA3 during the delay period in rats in a spatial position short-term memory task (Hampson et al., 2000) and in monkeys in an object–place and a location–scene association short-term memory task (Watanabe and Niki, 1985; Rolls, 1989a; Colombo et al., 1998; Wirth et al., 2003).

In sum, these results strongly suggest that rapid, plastic changes in the CA3a,b network are essential in encoding novel information involving associations between objects and places, odors and places, or between landmark visual cues and spatial locations, and that NMDA receptor-mediated plasticity mechanisms appear to play a significant role in the process.

### PAIRED-ASSOCIATE LEARNING

It has been suggested that the hippocampus and its subregions support associative processes as observed in paired-associate learning tasks (Eichenbaum and Cohen, 2001). McNaughton and Morris (1987), Hasselmo and Wyble (1997), and Rolls (1996) have suggested that the hippocampus and specifically the CA3a,b auto associative network is responsible for the formation and storage of associations. For example, information from parietal cortex regarding the location of an object may be associated with information from temporal cortex regarding the identity of an object. These two kinds of information may be projected to the CA3 a,b region of the hippocampus via the medial and lateral perforant path to enable the organism to remember a particular object and its location. Support for this idea comes from the observation that under the influence of direct infusions into the CA3a,b of AP5 (an NMDA antagonist), which should block transmission of spatial information from the medial perforant path, or naloxone (a μ opiate antagonist), which should block transmission of object information from the lateral perforant path (Breindl et al., 1994), naloxone or AP5 infusions into the CA3a,b disrupted both novelty detection of a spatial location and a visual object (Hunsaker et al., 2007c).

In order to directly test the involvement of the CA3a,b subregion of the hippocampus in spatial paired-associate learning, rats were trained on a successive discrimination go/no-go task to examine object–place paired-associate learning. In this task, two paired-associates were reinforced which consisted of one particular object (A) in one particular location (1) and a different object (B) in a different location (2). Mispairs that were not reinforced included object (A) in location (2) and object (B) in location (1). Rats should learn that if an object was presented in its paired location then the rat should displace the object to receive a reward (Go). However, the rat should withhold displacing the object if it was not in its paired location (no-go). The results indicate that rats with CA3a,b lesions were impaired in learning object–place paired-associations relative to controls (Gilbert and Kesner, 2003).

In a second task, rats were trained on a successive discrimination go/no-go task to examine odor–place paired-associate learning. In this task, the same procedure was used, except that rats needed to learn that when an odor was presented in its paired location the rat should dig in sand mixed with the odor to receive a reward. The results indicated that rats with CA3a,b lesions were severely impaired relative to controls in learning odor–place paired-associations (Gilbert and Kesner, 2003). However, it should be noted that animals with DG or CA1 lesions learned the object–place or odor–place task as well as controls (Gilbert and Kesner, 2003). Similar impairments were obtained following deletion of CA3 NMDA receptors in mice in the acquisition of an odor–context paired-associate learning task (Rajji et al., 2006). These data support the hypothesis that CA3a,b, but not DG or CA1, support paired-associate learning when a stimulus must be associated with a spatial location. To test this idea further, rats were given CA1, CA3a,b, or control lesions prior to learning an object–trace–odor task. The task was run in a 115-cm linear box in which the rat was presented with an object for 10 s, after which it was removed, followed by a 10-s trace period and by the presentation of an odor 50 cm away. If the odor and the object were paired, the rat was to dig in the odor cup for a reward. If unpaired, the rat was to refrain from digging. Animals that had CA1 lesions were unable to make the association and never performed above chance, whereas animals that had CA3a,b lesions performed as well as controls (Kesner et al., 2005). These results support the idea that the CA1 is involved in forming associations that do not necessarily involve spatial information, as long as temporal processing is required. Thus, the CA1 appears to be critical for mediating associations with temporal components, whereas the CA3a,b is important for mediating associations that involve spatial components. In support of the above mentioned statement, it has been shown that both CA3a,b and CA1 lesions disrupt the acquisition of an object–trace–place paired-associate learning task, suggesting that the CA1 appears to be critical for mediating an association with a temporal component, whereas the CA3a,b is important for an association that involves a spatial component (Hunsaker et al., 2006).

Computationally, one hypothesis would be that the CA3 system could provide the working memory necessary for hippocampus-dependent associations across time, and that the CA3 then influences the CA1 for this function to be implemented. The actual learning could involve holding one item active in CA3 but continuing firing in an attractor state until the next item in the sequence arrives, when it could be associated with the preceding item by temporally asymmetric synaptic associativity (Rolls and Kesner, 2006). The computational suggestion thus is that associations across time could be implemented in the hippocampus by using the same functionality that may be used for sequence memory. This model can account for the CA3a,b and CA1 deficits in the object–trace–place paired-associate learning, but cannot account for the CA1, but not CA3a,b, deficit in the object–trace–odor paired-associate learning task. A different computational model to explain the role of the hippocampus in mediating arbitrary associations across time is the temporal context model described by Howard et al. (2005) and Howard and Natu (2005). They suggest that the hippocampus binds arbitrary associations supplied by the entorhinal cortex into a retrievable spatio-temporal context. If one assumes that this could occur via the CA3a,b network or via direct connections to CA1, then this model could account for a significant role of CA1 in mediating arbitrary associations across time, including object–trace–odor and object–trace–place associations. However, it should be noted that the temporal context model only addresses the function of the hippocampus as a whole and does not address the role of CA1 and its potential underlying network in mediating arbitrary associations across time.

### CUED RECALL AND ARBITRARY ASSOCIATIONS

Even though the studies above measure the acquisition of paired-associate information, the tasks do not address whether the learning is based on arbitrary associations, in part because the stimuli in the object–place task are integral and cannot be separated from each other. Furthermore, since the tasks are bidirectional discrimination tasks, they are, according to O’Reilly and Rudy (2001) and Morris (2007), likely based on the learning of conjunctions of the cues associated with the task implying that that the cues cannot easily be separated from each other. In the standard model (Marr, 1971; McNaughton and Morris, 1987; Levy, 1996; Hasselmo and Wyble, 1997; Rolls and Treves, 1998; Rolls and Kesner, 2006), the CA3 system acts as an autoassociation system. This enables arbitrary (especially spatial in animals and likely language for humans as well) associations to be formed within the hippocampus. The CA3 recurrent collateral associative connections enable bidirectional associations to be formed between whatever stimuli are represented in the hippocampus, in that, for example, any place could be associated with any object, and in that the object could be recalled with a spatial recall cue, or the place with an object recall cue (Rolls and Treves, 1998).

In one ingenious experiment, Day et al. (2003) trained rats in a study phase to learn in one trial an association between two flavors of food and two spatial locations. During a recall test phase they were presented with a flavor which served as a cue for the selection of the correct location. They found that injections of an NMDA receptor antagonist (APV) or α-amino-3-hydroxy-5-methyl-4-isoxazolepropionic acid (AMPA) receptor antagonist (6-cyano-7-nitroquinoxaline-2,3-dione, CNQX) to the dorsal hippocampus prior to the study phase impaired encoding, but injections of APV prior to the test phase did not impair place recall, whereas injections of CNQX did impair place recall. The interpretation is that, in the hippocampus, NMDA receptors are necessary for forming one-trial flavor–place associations, and that recall can be performed without further involvement of NMDA receptors. The reverse order of cuing the location to recall the flavor has not been tested, so that one cannot be sure whether the hippocampus supports arbitrary associations based on this set of experiments.

Based on the Day et al. (2003) experiment, in the Kesner laboratory, a visual object recall for a spatial location task has been developed. In this task, after training to displace objects, rats in the study phase are placed in the start box and when the door in front of the start box is opened the rats are allowed to displace one object in one location, and then after returning to the start box, the door is opened again and the rats are allowed to displace a second object in another location. There are 50 possible objects and 48 locations. In the test phase, the rat is shown one object (first or second randomized) in the start box as a cue, and then, after a 10-s delay, the door is opened and the rats must go to the correct location (choosing and displacing one of two identical neutral objects). The rats receive a reward for selecting the correct location that was associated with the object cue. A spatial location-recall for a visual object task has also been developed. For the spatial recall for a visual object task, the study phase will be the same, but in this case in the test phase when the door is opened the rat is allowed to displace a neutral object in one location (first or second randomized) on the maze as a location cue, return to the start box, and then, after a 10-s delay, the door is opened and the rats must select the correct object (choosing and displacing one of two visual objects). The rats received a reward for selecting the correct visual object that was associated with the location cue. Rats learn both tasks with 75% or better accuracy. Results indicate that CA3a,b lesions produce chance performance on both the one-trial object–place recall and the place–object recall task (Kesner et al., 2008). The potential implications of such results are that indeed the CA3a,b supports arbitrary associations as well as episodic memory based on one-trial learning. A control fixed visual conditional to place task with the same delay was not impaired, showing that it is recall after one-trial (or rapid) learning that is impaired. Additional support comes from the finding that in a similar one-trial object–place learning followed by recall of the spatial position in which to respond when shown the object, Rolls et al. (2005) showed that some primate hippocampal (including especially CA3a,b) neurons respond with increased activity in the correct spatial location during and after the recall object cue. Thus, some hippocampal neurons appear to reflect spatial recall given an object recall cue. These data are consistent with the prediction of the standard computational model which emphasizes the importance of CA3a,b in mediating the development of arbitrary associations.

There is anatomical support for CA3a,b involvement in support of the mediation of associative processes including arbitrary associations. The perforant path from the entorhinal cortex can be divided into a medial and lateral component. It has been suggested that the medial component processes spatial information and that the lateral component processes non-spatial (e.g., objects, odors) information (Witter et al., 1989; Hargreaves et al., 2005). In one study, Ferbinteanu et al. (1999) showed that lesions of the medial perforant path disrupted water maze learning, whereas lateral perforant path lesions had no effect. In a more recent study based on the idea that the medial perforant path input into the CA3 mediates spatial information via activation of NMDA receptors and the lateral perforant path input into the CA3 mediates visual object information via activation of opioid receptors, the following experiment was conducted. Using a paradigm developed by Poucet (1989), rats were tested for the detection of a novel spatial change and the detection of a novel visual object change under the influence of direct infusions of AP5 (an NMDA antagonist) or naloxone (a μ opiate antagonist) into the CA3a,b. The results indicated that naloxone or AP5 infusions into the CA3a,b disrupted both novelty detection of a spatial location and a visual object (Hunsaker et al., 2007c). Based on electrophysiological data, there is associative long-term potentiation (LTP) between the medial or lateral perforant path and the intrinsic commissural/associational–CA3 synapses, demonstrated by the finding of an associative (cooperative) LTP between the medial and lateral perforant path inputs to the CA3 neurons (Martinez et al., 2002). This could provide a mechanism for object (via lateral perforant path)–place (via medial perforant path) associative learning, with either the object or the place during recall activating a CA3 neuron. Either place or object recall cues could thus be introduced by the associative medial and lateral perforant path connections to CA3 cells.

It should be noted that in contrast to the results obtained with a CA3a,b injection of naloxone or AP5, naloxone infusions into the DG disrupted both novelty detection of a spatial location and a visual object, whereas AP5 infusions into the DG disrupted only detection of a novel spatial location, but had no effect on detection of a novel object. Furthermore, infusions of AP5 into the CA1 region disrupts only the detection of a spatial location change, but not a visual object change, whereas naloxone into the CA1 region disrupts only the detection of a visual object change, but not a spatial location change (Krug et al., 2001; Hunsaker et al., 2007c). Thus, input pathways from medial and lateral perforant path have a different effect on each subregion in part based on the computational processes carried out by that specific subregion.

### PATTERN COMPLETION

During retrieval of information, Marr (1971) suggested that the hippocampus recurrent collaterals should play a major role in the hippocampus in retrieving originally stored information patterns in the face of partial inputs to the hippocampus (“collateral effect” or pattern completion). McNaughton and Morris (1987) and Rolls and Treves (1998) suggested that an autoassociative network within CA3 should be able to support pattern completion. Experimental efforts to find evidence of pattern completion within the CA3 region have been successful in recent years. Support for the pattern completion process in CA3 can be found in lesion studies. In one study, Nakazawa et al. (2002) trained CA3 NMDA receptor-knockout mice in the standard water maze task. When the animals were required to perform the task in an environment where some of the familiar cues were removed, they were impaired in performing the task. The result suggests that the NMDA receptor-dependent synaptic plasticity mechanisms in CA3 are critical to perform the pattern completion process in the hippocampus. In a different study, Fellini et al. (2009) reported that with the use of the water maze, NMDA receptors were critical for long-term memory retrieval in pattern completion. In another study (Gold and Kesner, 2005), rats were tested on a cheese board with a black curtain with four extramaze cues surrounding the apparatus. (The cheese board is like a dry land water maze with 177 holes on a 119-cm diameter board.) A Plexiglas partition, 7 cm deep and 8 cm tall, with a 7-cm opening to permit the animal access to each individual food well was placed on the 10th row of the cheese board. Rats were trained to move a sample phase object covering a food well that could appear in one of five possible spatial locations. During the test phase of the task, following a 30-s delay, the animal needs to find the same food well in order to receive reinforcement with the object now removed. Without the Plexiglas partition, rats would adopt a strategy of leaving the goal box directly to the center of the maze and make a right or left turn across the line of potential locations of the reward. The Plexiglas partition discouraged rats from using this strategy and forced the animal to make a clear location choice on the test phase by entering the partition surrounding a particular food well. The rats could not see the food until they made a choice. After reaching stable performance in terms of accuracy to find the correct location, rats received lesions in of dorsal CA3a,b. During post-surgery testing, four extramaze cues were always available during the sample phase. However, during the test phase, zero, one, two, or three cues were removed in different combinations. The results indicate that controls performed well on the task regardless of the availability of one, two, three, or all cues, suggesting intact spatial pattern completion. Following the CA3a,b lesion, however, there was an impairment in accuracy compared to the controls especially when only one or two cues were available, suggesting impairment in spatial pattern completion in CA3a,b-lesioned rats (Gold and Kesner, 2005). A useful aspect of this task is that the test for the ability to remember a spatial location learned in one presentation can be tested with varying number of available cues, and many times in which the locations vary, to allow for accurate measurement of pattern completion ability when the information stored on the single presentation must be recalled. In a subsequent study, naloxone which blocks μ opioid receptors or AP5 which blocks glutamate NMDA receptors were injected into the CA3 a,b region resulted in a disruption visual–spatial pattern completion, whereas AP5 did not disrupt visual–spatial pattern completion, but instead inhibited short-term or working memory (Kesner and Warthen, 2010).

In a different study, Vazdarjanova and Guzowski (2004) placed rats in two different environments separated by ~30 min. Different objects were located in two different environments. The authors were able to monitor the time course of activations of ensembles of neurons in both CA3 and CA1, using a new immediate-early gene-based brain-imaging method (Arc/H1a catFISH). When the two environments were only modestly different, CA3 neurons exhibited higher overlap in their activity between the two environments compared to CA1 neurons. In the early physiological studies, Robertson et al. (1998) and Rolls (1996) showed that some cells in CA3 respond when a monkey’s view of a specific part of space is obscured by a curtain or darkness creating an absence of specific visual inputs. Similar findings have been reported in rats (Samsonovich and McNaughton, 1997). In a more recent study Lee et al. (2004) recorded from ensembles of neurons using multiple electrodes in both CA1 and CA3a,b in freely behaving animals. By altering cue configuration in a ring track, Lee et al. (2004) demonstrated that modestly altered cue configurations was less disruptive in the population spatial code in CA3a,b. However, the population representation of space in CA1 was more easily disrupted by such moderately altered cue configurations. This can be considered as a pattern completion process in CA3a,b, since the CA3a,b network maintained similar spatial representation of the environment (compared to the original spatial representation of the familiar cue configurations) even though the relationships among cues in the environment were altered.

In general, there is good agreement as to the functions of the CA3. Differences emerge in determining whether the CA3 stores long-term episodic information (Rolls and Treves, 1998) that would aid in the pattern completion process or that the neocortex stores long-term episodic information that is transmitted to the CA3 region via the entorhinal cortex and the perforant path (Hunsaker and Kesner, 2013). Most likely the CA3 would employ both sets of information in the process of supporting pattern completion. Two sets of data support the influence of the perforant path into CA3 in that naloxone which blocks μ opioid receptors injected into the CA3 disrupts pattern completion (Kesner and Warthen, 2010) and lesions of the perforant path input into CA3 disrupts retrieval of newly learned information based on previous day learning of the Hebb–Williams maze (Lee and Kesner, 2004b).

Does the dorsal CA3a,b region also play a role in completing a previously learned fixed sequence of spatial locations when the rats are placed in any start position other than the first position and thereby supporting temporal–spatial pattern completion? In one study, Olton et al. (1984) trained rats on fixed sequence in an eight-arm maze based on the magnitude of the reward available in each of four arms (18, 6, 1, or 0 sucrose pellets). After learning the sequence and with some experience of starts in a different arm of the sequence, the rats were given fimbria-fornix or control lesions. The control rats performed the sequence with no difficulty even when new start arms were used, demonstrating temporal–spatial pattern completion. The fimbria-fornix lesioned rats made errors by always returning to the first position in the sequence regardless of which start position was used. This suggests that fimbria-fornix lesioned rats cannot remember the correct temporal–spatial context, but they do not have any difficulty remembering the serial order of the sequence.

In a more recent study, DeCoteau and Kesner (2000) trained rats in a sequential spatial location list learning paradigm using a correction procedure to visit the arms of an eight-arm maze in a particular sequence, with food obtained in each arm if a correct choice (made by orienting) was made. Even though lesions of the hippocampus impaired the acquisition of this task, there were no deficits when hippocampus lesions were made after reaching 90–100% correct performance (DeCoteau and Kesner, 2000). Thus, the hippocampus may be necessary for learning new spatial sequences, but once learned, the hippocampus lesioned rats can perform the sequence as long as the rats are started from the beginning of the sequence. To test for subregional specificity, control, dorsal CA3a,b, and dorsal CA1 lesioned rats were tested postoperatively for completion of the sequence when started in different positions in a previously learned list of spatial locations (Hoang and Kesner, 2008). The results indicate that control and dorsal DG lesioned rats had no difficulty completing the sequence, regardless of starting point. In contrast, when the dorsal CA3a,b lesioned rats were started in different positions within the sequence, they would invariably orient in front of the first position before making other response sequence errors. On a quantitative level when they made an orienting response to the wrong door (error) in the sequence, the average probability of making incorrect orienting responses for each position indicated that the dorsal CA3a,b rats made many incorrect orienting responses (errors). This suggests that CA3a,b lesioned rats cannot remember the correct temporal–spatial context implying difficulty in temporal–spatial pattern completion (Hoang and Kesner, 2008). These data are consistent with the observation that fimbria-fornix lesioned rats always returned to the beginning of the first position in the sequence regardless of which start position was used (Olton et al., 1984), even though it should be noted that in the Olton et al. (1984) study, the list length was shorter, the list sequence was influenced by magnitude of reinforcement, and the lesioned rats completed the sequence without making many errors. In the same study, when the dorsal CA1 lesioned rats were started in different positions within the sequence, they would orient in front of any of the forward located positions suggesting the use of random orienting responses (errors) across the completion of the sequence. On a quantitative level when they made an orienting response to the wrong door (error) in the sequence, the average probability of making incorrect orienting response indicated that CA1 lesioned rats made decreasing numbers of incorrect orienting responses as the starting position is increased. The errors made by CA1 lesioned rats appeared to be due to difficulty in temporal spatial pattern completion in that the decrease in errors was due to the observation that all the errors were made in the process of completing the spatio-temporal sequence, suggesting that CA1 lesioned rats have difficulty in remembering the serial order of the spatial sequence. These results suggest that the dorsal CA3a,b in conjunction with CA1 supports recall of a temporal sequence of spatial locations, requiring a completion process. Both CA3a,b and CA1 are likely involved because CA3a,b processes the spatial–temporal context and CA1 processes temporal information. From a computational point of view it has been suggested that temporally asymmetric associations between the successive pairs of items in a sequence are implemented in dorsal CA3 by some temporally asymmetric synaptic modifiability (Levy, 1996; Mehta et al., 2002; Howard et al., 2005; Jensen and Lisman, 2005) and the dorsal CA1 network in this situation could help to separate out the representation of the next item in the list from the currently processed item via a chunking process and thus providing for a temporal–spatial context suggesting that the dorsal CA1 can be thought of as performing temporal pattern completion for spatial locations (Kesner et al., 2004; Rolls and Kesner, 2006). These models are consistent with the evidence that rats with hippocampus lesions do not learn the fixed spatial sequence task (DeCoteau and Kesner, 2000). Alternatively, Lisman (1999), Lisman and Otmakhova (2001), and Lisman and Grace (2005) have suggested that the hippocampus operates a match–mismatch detector during the processing of novel changes within a sequence or start of a sequence. They suggest that the first spatial location can serve as a trigger for recall of the entire sequence within the dorsal CA3 region, which would be consistent with the errors made by CA3a,b lesioned rats which involved an error for the first position regardless of which start position was used. Because the readout is presumed to be time compressed, it reaches dorsal CA1 more quickly allowing the dorsal CA1 region to anticipate what will happen next based on previous experience. Based on the idea that the dorsal CA1 operates as a match–mismatch detector comparing predictions arriving from dorsal CA3a,b compared to direct entorhinal inputs into dorsal CA1 (Hasselmo and Schnell, 1994; Hasselmo and Wyble, 1997), the dorsal CA1 should play an important role in detection of changes in the sequence, which is consistent with the observation that lesions of the dorsal CA1 region results in errors that appeared to be random. Thus, the hippocampus and more specifically the dorsal CA1 and dorsal CA3a,b subregions contribute to the processing of previously learned sequence information for spatial locations and most likely contribute to the establishment of learned sequences.

### A SPECIAL ROLE FOR CA3c

It has been suggested that CA3 might also play a role in spatial pattern separation, but in this case not for a metric spatial representation, but for spatial representation of the geometry of the environment. This idea is supported by the finding of Tanila (1999) who showed that CA3c place cells were able to maintain distinct representations of two visually identical environments, and selectively reactivate either one of the representation patterns depending on the experience of the rat. Also, Leutgeb et al. (2005, 2007) recently showed that when rats experienced a completely different environment, CA3c place cells developed orthogonal representations of those different environments by changing their firing rates between the two environments, whereas CA1 place cells maintained similar responses. One model described by Rolls and Kesner (2006) to account for these results proposes that the CA3 and DG may have similar representations of the environment. This may be consistent with the computational point of view that if dorsal CA3 is an autoassociator, the pattern representations in it should be as orthogonal as possible to maximize memory capacity and minimize interference. The theory holds that the actual pattern separation may be performed as a result of the operation of the dentate granule cells as a competitive net, and the nature of the mossy fiber connections to dorsal CA3 cells. To test this idea an experiment was conducted to determine whether the DG or CA3 regions cooperate to perform spatial pattern separation operations for specific spatial locations as well as the spatial geometry of the environment or whether the DG performs spatial pattern separation on the basis of specific locations in space and the CA3 performs spatial pattern separation on the basis of the geometry of the environment. Rats with lesions of DG and CA3a,b were given the opportunity to explore a white or black circular or square box of the same size as reported by Leutgeb et al. (2007) and in addition in the box there were two objects spaced 68 cm apart. After habituation to the box and the objects, the rats received one of two transfer tests. In the first test, the objects were changed to a 38 cm distance, but the box shape (geometry of the environment) remained the same (Hunsaker et al., 2008a). In the second test, the box shape (geometric environment) was changed, but the distance between the objects remained the same. The efficacy of the transfer test in terms of re-exploration of the metric change is based on a comparison between the level of object exploration during the transfer session versus the level of object exploration during the last session of habituation. Similarly, the efficacy of the transfer test in terms of re-exploration of the geometry of the environment is based on the number of grid crossings (activity level) and rearings during the transfer session versus the number of grid crossings and rearings during the last session of habituation. The results indicate that lesions of the DG, but not CA3a,b, disrupt both the detection of metric changes in the spatial location of objects and changes in a geometrical environment. Thus far, these data are consistent with the prediction of the Rolls computational model that the DG is the critical substrate for spatial pattern separation. These data are not consistent with (Tanila, 1999; Leutgeb et al., 2005, 2007) findings of a pattern separation function for geometrical environments. It has been shown that the CA3 region can be divided into a CA3a, b, and c subareas (Lorente de Nó, 1934; Li et al., 1994). Most of the recording of cells that respond to different environments reported by Tanila (1999) and Leutgeb et al. (2005, 2007) were based on electrode placements in the CA3c area. The present lesion data are based on lesions within CA3a,b, but not CA3c. Based on the research of Li et al. (1994) and Buckmaster and Schwartzkroin (1994), it has been proposed that mossy cells receive excitatory inputs from granule cells and CA3c pyramidal cells and integrate the inputs from granule cells and CA3c pyramidal cells, which, in turn, via excitatory recurrent axonal projections activate many distal granule cells. Such a circuit could integrate spatial location information and form representations of geometrical environments. Additional experiments with CA3c lesions in contrast to the CA3a,b lesions revealed that dorsal CA3c is only involved in pattern separation processes when the animal is required to detect a metric change in object location. This was observed during the metric change in object location task (with both decreased object exploration and rearing; but at a smaller magnitude than the DG effect). There was no apparent effect of a CA3c lesion during the environmental change task. To explain these results, it may be that the DG selectively recruits CA3c to assist in the metric detection and not the detection of the overall environmental change. This interpretation is in agreement with the suggestions of Scharfman (2007), who suggests that CA3c cooperates with the DG to support efficient spatial memory formation and subsequent recall and is supported by Jinde et al. (2012) and Jinde et al. (2013), who demonstrated that hilar mossy cells, receiving CA3c inputs, play an overall inhibitory role in DG excitability. Also, it is possible that if CA3c forms a circuit with the DG that participates in sparse encoding of entorhinal inputs, the feedback circuit would be more critical for detection of discrete changes, such as after metric shifts in object locations. Since the change in overall environmental geometry is a very large and prominent change, the feedback circuit may not be as relevant due to the size of the change (i.e., the DG can easily orthogonalize the two environments with or without CA3c feedback modulation). The implication of this theory is that for small-scale or subtle changes, the dorsal DG and dorsal CA3c cooperate to orthogonalize the incoming visuo-spatial data with a fair degree of resolution. The CA3c projection to the hilar mossy cells would act to inhibit the granule cells, facilitating even sparser encoding than the DG would normally provide (i.e., a more orthogonal representation). For larger changes, such as for an overall environmental change, CA3c is not needed because the requirement for sparse encoding is not present (low spatial resolution is more than sufficient to distinguish a circle from a square environment). That is to say, it is easier for the animal to identify orthogonal charts than to process metric relationships between stimuli (Rolls and Kesner, 2006).

## FUNCTIONAL ANALYSIS OF THE CA3 SCHAFFER COLLATERAL INPUTS INTO CA1

The CA3 has a direct output pathway into CA1, suggesting the possibility of CA3 modulation of CA1 functions. Rolls and Kesner (2006) have suggested that the CA1 has at least four major functions, including temporal processing of information (temporal order memory), associations across time, intermediate memory, and consolidation of new information. One of the mnemonic processes suggested for CA1 involves the mediation of temporal processing of information involving chunking and temporally separating information to endow spatial and/or non-spatial contexts with a temporal structure of the information to be remembered. This process would require extensive processing of information in CA1 and thus may influence intermediate-term rather than short-term memory representations. If one assumes that CA3 is important for associating, processing, and integrating sequential information, and perhaps for maintaining a short-term memory representation of sequentially associated information as a context, then this information might be sent via feed-forward connections to CA1. Computational models and physiological data (Skaggs et al., 1995; Rolls, 1996) have suggested that CA1 may play a role in compressing temporal sequences. Rolls and Kesner (2006) have suggested that CA1 recodes the information represented in the CA3 network by holding one item active in CA3, but continuing firing in an attractor state until the next item in the sequence arrives, when it can be associated with the preceding item by temporally asymmetrical synaptic associativity, possibly through a chunking process.

### TEMPORAL ORDER MEMORY

It has been shown in humans that order judgments improve as the number of items in a sequence between the test items increases (Banks, 1978; Chiba et al., 1994; Madsen and Kesner, 1995). This phenomenon is referred to as a temporal distance effect (sometimes referred to as a temporal pattern separation effect; Kesner et al., 2004). The temporal distance effect is assumed to occur because there is more interference for temporally proximal events than for temporally distant events. To test for the temporal pattern separation effect in rodents, Gilbert et al. (2001) tested memory for the temporal order of items in a one-trial sequence learning paradigm in rodents. In the task, each rat was given one daily trial consisting of a sample phase followed by a choice phase. During the sample phase, the animal visited each arm of an eight-arm radial maze once in a randomly predetermined order and was given a reward at the end of each arm. The choice phase began immediately following the presentation of the final arm in the sequence. In the choice phase, two arms were opened simultaneously and the animal was allowed to choose between the arms. To obtain a food reward, the animal had to enter the arm that occurred earlier in the sequence that it had just followed. Temporal separations of 0, 2, 4, and 6 were randomly selected for each choice phase. These values represented the number of arms in the sample phase that intervened between the arms that were to be used in the test phase. After reaching criterion, rats received control, CA3 or CA1 lesions. The results indicated that control rats matched their preoperative performance across all temporal separations. In contrast, rats with CA1 lesions performed at chance across zero, two, or four temporal separations and a little better than chance in the case of a separation of six items. The results suggest that the CA3 and CA1 subregions are involved in memory for spatial location as a function of temporal separation of spatial locations, suggesting that lesions of the CA3 and CA1 decrease efficiency in temporal pattern separation. Using the same paradigm as described above for odors resulted in intact function for the dorsal CA1, but a deficit was observed for ventral CA1 (Kesner et al., 2010). There are no data available to suggest that the ventral CA3 may also produce a deficit. These results suggest CA3 can influence CA1 function and perhaps promote an inability to inhibit interference that may be associated with sequentially occurring events. The increase in temporal interference impairs the rat’s ability to remember the order of specific events. Although CA1 lesions have been shown to produce a deficit in temporal pattern separation (Gilbert et al., 2001), some computational models (Levy, 1996; Wallenstein and Hasselmo, 1997; Lisman, 1999; Rolls and Kesner, 2006) have suggested that the CA3 region is an appropriate part of the hippocampus to form a sequence memory, for example, by utilizing synaptic associativity in the CA3–CA3 recurrent collaterals that entail a temporally asymmetrical component.

In a more recent experiment using an exploration paradigm described by Hannesson et al. (2004), it can be shown that temporal order information for visual objects is impaired only for CA1, but not for CA3 lesions (Hoge and Kesner, 2007). Thus, based on previous research, it has been suggested that the CA1 hippocampal subregion serves as a critical substrate for sequence learning and temporal order or temporal pattern separation. This position would be consistent with CA1 deficits in temporal order for spatial locations, odor, and visual objects (Hunsaker et al., 2008c). In addition, it appears that dorsal CA3 contributes to this temporal order sequential process whenever spatial location or odor are a factor; however, the CA3 region does not appear to play a role in these processes for visual object information (Hoge and Kesner, 2007; Hunsaker et al., 2008c). It is important to note that selective lesions of the NR1 subunit of the NMDA receptor in CA3 or CA1 resulted in a lack of any effect on temporal processing using the spontaneous exploration paradigm, which suggests that the NMDA receptor does not play an important role in temporal processing (Place et al., 2012).

## ASSOCIATIONS ACROSS TIME

There is evidence implicating the hippocampus in mediating associations across time (Rawlins, 1985; Kesner, 1998). In particular, the CA1 subregion of the hippocampus may play a role in influencing the formation of associations whenever a time component (requiring a memory trace) is introduced between any stimuli that need to be associated. It has previously been shown that the acquisition of an object–odor association is not hippocampus-dependent (Gilbert and Kesner, 2003); however, it is unclear whether adding a temporal component would recruit the hippocampus and more specifically the CA1 region. To test this idea, rats were given CA1, CA3, or control lesions prior to learning an object–trace–odor task (Kesner et al., 2005). The task was conducted in a 115-cm linear box, in which the rat was presented with an object for 10 s, after which it was removed, followed by a 10-s trace period and by the presentation of an odor 50 cm away. If the odor and the object were paired, then the rat was to dig in the odor cup for a reward. If the odor and the object were unpaired, then the rat was to refrain from digging. Animals that had CA1 lesions were unable to make the association and never performed above chance, whereas animals that had CA3 lesions performed as well as control rats (Kesner et al., 2005). Furthermore, it can also be shown (MacDonald et al., 2011) that in the same paradigm neuronal activity from CA1 neurons fire during the trace interval reflecting location and ongoing behavior, which could also be based on the anticipation of the odor component of the task. However, no recordings were made of neurons within CA3. These results support the idea that the CA1 is involved in forming arbitrary associations that do not necessarily involve spatial information, as long as temporal processing is required. In support of this conclusion, it has been shown that both CA3 and CA1 lesions disrupt the acquisition of an object–trace–place paired-associate learning task, suggesting that the CA1 appears to be critical for mediating an associations with a temporal component, whereas the CA3 appears to contribute to temporal associations only when a spatial component is involved (Hunsaker et al., 2006). In an interesting study, McEchron et al. (2003) recorded from single cells in the CA1 region of the hippocampus during and after trace heart rate (fear) conditioning using either a 10- or 20-s trace interval. They reported that a significant number of cells showed maximal firing on CS-alone retention trials timed to the 10- or 20-s trace after CS offset. This finding could reflect the transition from an attractor state implemented in CA3 with its recurrent collaterals that represents the CS to an attractor state that represents the conditioned response (CR; Rolls and Deco, 2002; Deco and Rolls, 2003, 2005). Computationally, one hypothesis would be that the CA3 system could provide the short-term memory necessary for hippocampus-dependent associations across time, and that the CA3 then influences the CA1 for this function to be implemented. The actual learning could involve holding one item active in CA3, but continuing firing in an attractor state until the next item in the sequence arrives, at which point it could be associated with the preceding item by temporally asymmetrical synaptic associativity (Rolls and Kesner, 2006). The computational suggestion is thus that associations across time could be implemented in the hippocampus by using shared functionality that may be used for sequence memory.

## INTERMEDIATE MEMORY

In addition to the processing of temporal information, the CA1 subregion appears to entail cellular processes that match it to longer-term types of memory (intermediate-term memory) than that of CA3, which is thought to mediate short-term memory. To examine this idea rats with either CA3 or CA1 lesions were tested on a DNMP task in an eight-arm maze with a 10-s delay. Animals with CA3 lesions showed reliable acquisition impairments at the 10-s delay. Animals with CA1 lesions showed normal acquisition, suggesting that the CA1 region may not be necessary at short delay intervals (Lee and Kesner, 2003). Moreover, when transferred to a new maze in a different room and using a 10-s delay, similar impairments were observed for CA3- but not CA1-lesioned rats. Deficits for CA1 did not emerge until a 5-min delay was introduced. Interestingly, comparable deficits at a 5-min delay were also found for CA3-lesioned rats (Lee and Kesner, 2003). It should be noted that similar results as described for the lesion data were obtained following AP5 injections, which impaired transfer to the new environment when made into CA3, but not into CA1. At 5-min delays, AP5 injections into CA1 produced a sustained deficit in performance, whereas AP5 injections into CA3 did not produce a sustained deficit (Lee and Kesner, 2002, 2003). In a different study using a continuous recognition task for odors, Farovik et al. (2009) reported that with a 3-s interval between odors, rats with lesions of the dorsal CA3, but not dorsal CA1 disrupted memory for the order of odor information, but with a 10-s interval between odors, both CA1 and CA3 lesioned rats were impaired for odor information.

### CONSOLIDATION AND LONG-TERM MEMORY

It has been suggested that the CA3 can influence the activity of CA1 by promoting a consolidation process resulting in long-term memory. To test this idea that the CA1 and CA3 region might be involved in retrieval after long (24 h) time delays, rats with CA1 or CA3 lesions were tested in a modified Hebb–Williams maze. The results indicated that CA3 lesioned rats were impaired in encoding (within-day tests), but with no deleterious effect on retrieval with a 24-h delay (across-day tests). In contrast, CA1-lesioned rats were impaired in retrieval with a 24-h delay (across-day tests), but they have no difficulty in encoding new information (i.e., within-day tests; Jerman et al., 2006; Vago et al., 2007). Using a spatial contextual fear-conditioning paradigm, rats with dorsal CA1, but not dorsal CA3, lesions were impaired relative to controls in retention (long-term memory) of contextual fear-conditioning (Lee and Kesner, 2004a,b; Hunsaker and Kesner, 2008). In a different study, Gall et al. (1998) demonstrated that c-fos gene expression was elevated selectively more in CA1 compared to CA3 only after overtraining in an olfactory discrimination task, whereas the same gene expression in CA3 compared to that in CA1 was selectively enhanced after the initial acquisition of the olfactory discrimination task. Furthermore, sustained neural activity over intervals of hours to days potentially promoting consolidation is mediated by CA1, but not CA3 neurons (Mankin et al., 2012). In a different task, Florian and Roullet (2004) showed that focal injections of diethyldithiocarbamate (DDC) into the CA3 region impaired the acquisition, but not recall of spatial information in the water maze. Also, injection of DDC into the CA3 region immediately after training in the water maze disrupted consolidation 24 h later. In a different study, Lassalle et al. (2000) also tested animals in the water maze and found that DDC injections into CA3 did not disrupt memory consolidation. The above-mentioned data suggest that the CA1 region may play an important role in supporting consolidation processes, but it appears that CA3 does not have a powerful modulatory effect on CA1 in affecting the consolidation process. Rather it is assumed that CA1 is important for consolidation of new information within the intermediate temporal memory framework by connecting with neocortical systems to promote consolidation.

## INTERACTIONS AND DISSOCIATIONS BETWEEN CA1 AND CA3

The dominant view of the relationship between CA3 and CA1 and short-term and intermediate-term memory is that they operate as a feed-forward sequential processing system. There is evidence to suggest that, under certain task conditions, both the CA1 and CA3 interact in the processing of short-term and intermediate-term memory (Gilbert et al., 2001; Lee et al., 2005; Kesner et al., 2008; Farovik et al., 2009). However, based on more recent data, it is suggested that, for certain tasks, dissociations exist between short-term and intermediate-term memory mediated by the CA3 and CA1 subregions, respectively (Kesner, 2007). Moreover, evidence of parallel processing between the CA3 and CA1 implies possible functional independence of short-term and intermediate-term memory processes. One result that has been obtained when one compares the relationships between CA3 and CA1 is that there is a deficit following dysfunction of the CA3 subregion, but no concomitant deficit following dysfunction of the CA1 subregion. For example, lesions of the CA3, but not the CA1, subregion impair the acquisition of the DNMP task on an eight-arm maze with 10-s delays (Lee and Kesner, 2003) or novelty detection of a spatial location (Lee et al., 2005). Furthermore, CA3, but not CA1, lesions impair within-day learning (encoding) in a Hebb–Williams maze (Jerman et al., 2006; Vago et al., 2007). Taken together, these results suggest that short-term and intermediate-term memory can operate independently of each other. Is there an anatomical basis for this apparent parallel operation between CA3 and CA1 and short-term and intermediate-term episodic memory? Given that CA1 represents the primary output from the hippocampus, especially in light of the fact that CA3 does not have direct axonal projections to the subiculum or entorhinal cortex, how can information be transmitted to other neural regions outside the hippocampus once CA1 is ablated? Evidence presented in more detail elsewhere indicates that there is an important output from the CA3 to the septum via the fimbria and that lesions of the fimbria can mimic the CA3 lesion effects (Hunsaker et al., 2007b, 2008b). Further evidence of a functional dissociation between CA1 and CA3 can be observed on a number of tasks that produce a deficit in CA1, but no concomitant deficit following dysfunction of CA3. For example, intermediate-term memory in a delayed (5-min) non-matching-to-sample for a spatial location task is disrupted by AP5 injections into the CA1, but not into the CA3 subregion (Lee and Kesner, 2002). Furthermore, temporal order for a visual object (Hoge and Kesner, 2007), retrieval of information acquired in a Hebb–Williams maze (Jerman et al., 2006; Vago et al., 2007), and retention of contextual fear-conditioning are disrupted by CA1 but not CA3 lesions (Lee and Kesner, 2004a; Hunsaker and Kesner, 2008). These results also suggest that intermediate-term and short-term memory can operate independently of each other, begging the question whether there is a different anatomical basis for this apparent parallel operation between CA3 versus CA1 and short-term versus intermediate-term memory. In all these cases when there is a deficit following CA1 lesions, but not CA3 lesions, the possibility exists that the deficit is due to a faulty input from the direct perforant pathway to CA1, because the Schaffer collateral input is intact.

Based on the idea that *in vitro* dopamine injections into the CA1 region inhibit the direct perforant path projection to the CA1 region without affecting the Schaffer collateral projection into the CA1 region (Otmakhova and Lisman, 1999), rats were injected with apomorphine (a non-selective dopamine agonist) or vehicle control in the CA1 region in the delayed (5 min) non-matching-to-sample for a spatial location task, in the Hebb–Williams maze task, and in the contextual fear-conditioning task. The results showed that apomorphine injections into the CA1 disrupted performance in all of these tasks. Furthermore, apomorphine injections into CA1 did not disrupt encoding in the Hebb–Williams maze or transfer of short-term memory for a spatial location in a non-matching-to-sample task to a novel environment (a new maze), which represent processes that are not dependent on the CA1 region (Vago et al., 2007; Vago and Kesner, 2008). Apomorphine injections into the CA1 subregion produce the same pattern of deficits as are produced by CA1 but not CA3 lesions. Taken together, these results suggest that in situations where there is a CA1 but no CA3 lesion effect, the direct perforant path input into the CA1 region represents the main input for the integrity of intact performance, perhaps based on intermediate-term memory processing.

It can also be shown that in some tasks, there is an interaction between CA3 and CA1 in that one can observe a deficit following dysfunction of either the CA3 or CA1 subregion. For example, when multiple temporally organized sequential information is to be remembered within a short-term memory system, such as memory for multiple spatial locations, both CA3 and CA1 lesions produce impairments (Gilbert et al., 2001; Lee et al., 2005; Kesner et al., 2008). Thus, there is the implication that both regions are working cooperatively and that CA1 is likely to benefit from a feed-forward Schaffer collateral connection from CA3.

## FUNCTIONAL ANALYSIS OF MOSSY FIBER VERSUS PERFORANT PATH INPUTS INTO CA3a,b

McNaughton and Morris (1987), Rolls and Treves (1998), and Rolls and Kesner (2006) have suggested that the dentate granule cell/mossy fiber pathway to CA3 may be important during the learning of new associations in the CA3 network, and that part of the way in which it is important is that it helps by pattern separation to produce relatively sparse and orthogonal representations in CA3. In addition, the theory proposes that the direct perforant path input to CA3 is important in initiating retrieval from the CA3 autoassociation network, especially with an incomplete retrieval cue.

In order to test the above mentioned ideas, a Hebb–Williams maze was used. In this task, rats were required to traverse a maze from the start to goal box in the most direct possible path. Learning across the final five trials of the first day compared to the first five trials of the first day of training was used as an index of encoding, and performance on the first 5 days of the second day compared to the last five trials of the first day was used as an index of retrieval. These operationally defined encoding and retrieval epochs were on average 10 min in length each. Using this task, it was found that lesions of the DG or CA3a,b (or a crossed lesion) disrupt within-day learning on the Hebb–Williams maze, but that retrieval of information at the start of the following day is not impaired (Lee and Kesner, 2004b; Jerman et al., 2006). In contrast, lesions of the perforant path input to CA3a,b from entorhinal cortex disrupt retrieval (i.e., initial performance on the following day), but not learning within a day (Lee and Kesner, 2004b). These findings support the hypothesis that the CA3 processes both inputs from the DG to support pattern separation and inputs from the entorhinal cortex via the perforant path to support pattern completion.

Furthermore, with respect to short-term memory, the DG and mossy fiber input into CA3 are not directly involved. This is illustrated by the observation that on a spatial pattern separation task, the performance of DG lesioned rats increased as a function of increased spatial separation between the target location and the foil location, whereas CA3 lesions were impaired across all spatial separations, indicating a potential impairment in short-term memory for spatial location information (Gilbert et al., 2001; Gilbert and Kesner, 2006).

Thus, from an anatomical perspective possible dissociations between DG and mossy fiber input into CA3 are primarily due to the observation that the CA3 subregion of the hippocampus has two major inputs with a direct connection from the DG via the mossy fibers and a direct input from the perforant path which bypasses the DG. In addition, short-term memory may be generated intrinsically in the CA3 region, but not in the DG.

### A MODEL FOR BIASING CA3 TOWARD PATTERN SEPARATION OR PATTERN COMPLETION

A circuit between the hippocampus and medial septum/diagonal band of Broca has been characterized, as well as an efferent pathway from the hippocampus to the lateral septum (Raisman et al., 1966; Swanson and Cowan, 1977, 1979; Wyss et al., 1980; Gaykema et al., 1991). Briefly, CA3 subcortical efferents in the fimbria terminate in the lateral septum, medial septum, and diagonal band of Broca that result in net medial septum/diagonal band of Broca inhibition. CA1 subcortical efferents in the dorsal fornix also terminate in the lateral septum, medial septum, and diagonal band of Broca that result in net medial septum/diagonal band of Broca excitation. These differential effects occur because the CA3 and CA1 projections synapse onto distinct neuron populations. The medial septum and diagonal band of Broca send cholinergic efferents via the fimbria into the hippocampus that have been implicated in the hippocampal theta rhythm and modulation of learning and memory (McLennan and Miller, 1974; Rawlins et al., 1979; McNaughton and Miller, 1986; Hasselmo and Bower, 1993; Hasselmo and Schnell, 1994; Hasselmo et al., 1995, 2002a,b; Hasselmo, 1999, 2005; Hasselmo and Fehlau, 2001; Hasselmo and McGaughy, 2004; Hasselmo and Giocomo, 2006). Critically, these studies also demonstrated that acetylcholine in the hippocampus acts presynaptically by inhibiting glutamate release, presumably through M4 muscarinic acetylcholine receptor activation primarily at synapses in the stratum radiatum. GABAergic inputs are involved in hippocampal function as well, but act too rapidly to modulate encoding and consolidation/retrieval as operationally defined in most studies (i.e., behavioral encoding and retrieval are measured in seconds or minutes as opposed to the millisecond time course of the GABAergic modulation within theta phase precession and similar processes; cf., Wallenstein and Hasselmo, 1997). It is suggested that these GABAergic projections are potentially involved in biasing pattern separation and pattern completion dynamics at millisecond timescales such as during theta cycles, and acetylcholine regulates these processes at longer time scales, such as the second to minute timescales encountered during cognitive testing. In CA1 and CA3, acetylcholine has a more robust inhibitory effect in stratum radiatum than stratum oriens, stratum lacunosum-moleculare or stratum lucidum in slice preparations. In the DG, there are dense cholinergic projections into the inner molecular layer as well as the hilus and acetylcholine effects to disinhibit granule neurons in the DG by reducing tonic inhibition.

The CA3 recurrent collaterals terminate primarily within the stratum radiatum, whereas the perforant path inputs terminate in the stratum lacunosum-moleculare and the mossy fibers terminate largely in the stratum lucidum with additional synapses onto thorny excrescences in the stratum oriens and stratum radiatum as well. Despite a partial overlap in connectivity among these input pathways, this pattern of connectivity suggests that the mossy fiber pathway (at least synapses in the stratum oriens and stratum lucidum) and perforant pathway inputs (in the stratum lacunosum-moleculare) are not as dramatically affected by acetylcholine influx as the recurrent collateral and Schaffer collateral inputs (which are presynaptically inhibited by acetylcholine via M4 receptors) – though they are bound to be affected to some degree, but this could arguably be beneficial and result in a high signal to noise ratio in the hippocampal system (cf., Hasselmo et al., 1995, 1996). These data suggest that acetylcholine modulates the hippocampus primarily by selectively altering the signal to noise ratio within the recurrent collaterals and Schaffer collaterals in the stratum radiatum by reducing synaptic transmission (Hasselmo and Schnell, 1994; Hasselmo et al., 1995, 1996).

An important additional effect of elevated acetylcholine levels in the hippocampus is the net result on DG granule neurons. Bilkey and Goddard (1985) demonstrated that activations of septal projections into the hippocampus resulted in disinhibition of granule neurons through inhibition of inhibitory interneurons that provide tonic inhibition to the granule neurons (i.e., disinhibition). Medial septal stimulation at magnitudes insufficient to result in evoked responses facilitated population spikes in the DG to medial perforant path stimulation *in vivo*. In other words, the tonic inhibition on the granule neurons from the inhibitory neurons in the hilus typically attenuating or shunting the activity levels of granule neurons is reduced by cholinergic influx – thereby increasing the levels of responsiveness to stimuli of the DG granule neurons under the same conditions that the influence of the recurrent collaterals in CA3 are minimized, supporting cholinergic models of encoding/retrieval dynamics in the hippocampus (cf., Hasselmo et al., 1996). Alternately, any reduction from the baseline levels of cholinergic influence in the DG would result in a net increase in inhibitory tone on granule neurons. This mechanism is important for models of hippocampal encoding and retrieval that require a relatively quiescent mossy fiber input to allow retrieval processes to be initiated via the perforant path projections from the entorhinal cortex (cf., Treves and Rolls, 1992, Treves and Rolls, 1994; Rolls, 1996).

It is important to note that the proposed mechanisms whereby the dentate gurus engages in pattern separation does not actually require low activity levels of granule neurons, but rather requires the sparseness of encoding – that is to say the divergent connections from the entorhinal cortex to the DG via the perforant path and the subsequent convergence of mossy fiber inputs to CA3 (Treves and Rolls, 1992, 1994; Rolls, 1996). In this case, the reduction of tonic inhibition in the DG would not result in reduced competitive inhibition, as the disinhibited granule neurons would be more likely to fire action potentials that excite the inhibitory interneurons that mediate competitive inhibition than they were when inhibited. What would be facilitated by disinhibition, however, is the responsiveness of the DG granule cells to perforant path inputs.

In other words, although the DG granule cells would be more responsive to entorhinal inputs, the cells would not sacrifice precision in their encoding because the following factors would remain unaltered: (1) the connectivity matrix with the entorhinal cortex would not be changed. (2) The competitive inhibition through local interneurons would be intact as the interneurons would still respond to mossy fiber inputs in the normal way. (3) CA3c is recruited in the dentate gurus network with the mossy cells in the hilus for pattern separation (cf., Myers and Scharfman, 2009, 2011) and these cells remain relatively unaffected by acetylcholine efflux (no recurrent collateral synapses in the stratum radiatum). When these three factors are not changed the net effect on the DG would actually be an increase in the overall competitive inhibition in the network but now with a lower threshold for initiation of these winner take all processes. This increased responsiveness to stimuli in the granule neurons, in concert with the reduced efficacy of the recurrent collateral synapses to activate CA3 pyramidal cells would facilitate information transfer from the mossy fibers to the CA3 pyramidal cells with no net sacrifice of DG-dependent pattern separation.

Based on the models proposed by Hasselmo and Bower (1993), it is suggested that CA1 provides a feedback control over the cholinergic modulation of the hippocampus. It is proposed that CA1 sends a “mismatch” signal to the lateral septum, medial septum, and diagonal band of Broca in response to information that needs to be encoded – a process facilitated by pattern separation. This means that if information from the Schaffer collateral fiber pathway does not match the information in the perforant path projections, then CA1 signals the septum to raise levels of acetylcholine in the hippocampus to facilitate pattern separation processes and attenuate pattern completion processes by reducing recurrent collateral transmission while disinhibiting the granule neurons in the DG. The CA1 projections, importantly, are excitatory on cholinergic projection cells, overriding any effects of CA3 projections in the fimbria that serve a somewhat tonic inhibitory function. Conversely, in the absence of a CA1 mismatch signal, CA3 projections via the fimbria to the medial septum and diagonal band of Broca result in reduced acetylcholine levels, which facilitate pattern completion and retrieval of learned patterns. This means that if information from the perforant path matches the information in the Schaffer collaterals, then CA1 does not send excitatory signals the septum, which when combined with CA3 projections that inhibit the medial septum, serves to attenuate levels of acetylcholine in the hippocampus to facilitate pattern completion and retrieval and attenuate pattern separation processes (for a quantitative analysis of the computational models being described; cf., Hasselmo and Schnell, 1994; Hasselmo et al., 1995, 1996). The effects on the DG of the reduced acetylcholine levels would be that the granule neurons would be inhibited and less responsive to perforant path stimulation from the entorhinal cortex, thus providing the necessary reduction in DG activity levels required for retrieval processes to occur per the models posited by Treves and Rolls (1992, 1994). Additional evidence for an important role for acetylcholine is based on the observation that GABAergic interneurons, such as oriens-lacunosum-moleculare (OLM), can alter plasticity in the Schaffer collateral pathway and can alter the operation of CA1 and CA3 by gating information flow in CA1, facilitating CA3 transmission to CA1 and reducing the influence of perforant path inputs (Leão et al., 2012).

It has been clearly demonstrated that disrupting CA3a,b subcortical efferents in the fimbria disrupts encoding, but not retrieval, of spatial information during learning of the Hebb–Williams maze task (Hunsaker et al., 2008b). Rats with CA3a,b outputs to the septal nuclei via the fimbria disrupted were impaired for encoding or within-day learning, but showed intact abilities to perform between day retrieval. Rats with CA1 outputs to the septal nuclei via the dorsal fornix/alveus disrupted showed intact abilities to perform within-day encoding but were impaired for between day retrieval. Importantly, for these tasks the cholinergic inputs to the hippocampus via the fimbria were intact.

It is suggested that this effect is due to a critical role for cholinergic inputs to the hippocampus in biasing CA3a,b to pattern separation via silencing the recurrent collateral influence relative to the mossy fibers, since a disruption of these cholinergic projections would disrupts the ability of CA3a,b to encode spatial information (Hunsaker et al., 2007a,b, 2008b, 2009). The consequence of increasing the cholinergic projections to CA3a,b would be a decrease in the synaptic transmission of the recurrent collaterals and thus less recurrent activation relative to the mossy fiber inputs. Also, when acetylcholine levels increase in the DG, the inhibitory interneurons in the hilus are inhibited – thus reducing the amount of tonic inhibition on the granule neurons. The effect of this disinhibition is to increase mossy fiber neurotransmission (assuming increased transmission follows from increased responsiveness to stimulation), further biasing CA3a,b toward encoding information from the DG over recurrent activity from the recurrent collaterals.

Reducing acetylcholine by attenuating excitation of the medial septum/diagonal band of Broca cholinergic neurons would favor pattern completion and recall over pattern separation and encoding and push CA3a,b into the role of a working memory buffer by reducing the signal to noise level, and thus providing the best environment for the detection and completion of patterns from partial cues (Kesner and Rolls, 2001). As mentioned above, concurrent with the effects on CA3, reducing acetylcholine would reduce inhibitory tone on inhibitory interneurons in the hilus – thus increasing inhibition on the DG granule neurons, and subsequently reducing mossy fiber inputs to CA3a,b. Furthermore models also support this assertion by demonstrating decreased acetylcholine levels in a septo-hippocampal classical conditioning learning model result in overall reductions in the learning rate (Myers et al., 1996).

The pattern of results for scopolamine (a cholinergic antagonist) and physostigmine (a cholinergic agonist) infusions into CA3a,b is intriguing. Scopolamine, but not physostigmine, infusions, into CA3a,b disrupt encoding. In contrast, physostigmine, but not scopolamine infusions, into CA3 a,b, disrupt recall. This was observed during a spatial exploration paradigm (Hunsaker et al., 2007b), during Hebb–Williams maze learning (Rogers and Kesner, 2003), and during delay fear-conditioning (Rogers and Kesner, 2004). In another study, Pereira et al. (2005) showed that injections of 192IgG-saporin in the hippocampus, which produced a severe depletion of cholinergic cells in the medial septum, was sufficient to disrupt acquisition of the Hebb–Williams maze. These data suggest that cholinergic levels directly influence information processing in the hippocampus, supporting the assertion that acetylcholine levels are perfectly located in time and place to bias CA3a,b toward either pattern separation or pattern completion.

## CONCLUSION

From a behavioral perspective the CA3a,b subregion of the hippocampus plays an important role in the encoding of new spatial information within short-term memory with a duration of seconds and minutes. This can easily be observed in tasks that require rapid encoding, novelty detection, one-trial short-term or working memory, and one-trial cued recall primarily for spatial information. These are tasks that have been assumed to reflect the operations of episodic memory and require interactions between CA3a,b and the DG via mossy fiber inputs into the CA3a,b. The CA3a,b is also important for encoding of spatial information requiring the acquisition of arbitrary and relational associations. All these tasks are assumed to operate within an autoassociative network function of the CA3 region. The CA3a,b also supports retrieval of short-term memory information based on a spatial pattern completion process. Based on afferent inputs into CA3a,b from the DG via mossy fibers and afferents from the entorhinal cortex into CA3a,b as well as reciprocal connections with the septum, CA3a,b can bias the process of encoding utilizing the operation of spatial pattern separation and the process of retrieval utilizing the operation of pattern completion. The CA3a,b also supports sequential processing of information in cooperation with CA1 based on the Schaffer collateral output from CA3a,b to CA1. The CA3c function is in part based on modulation of the DG in supporting pattern separation processes.

## Conflict of Interest Statement

The author declares that the research was conducted in the absence of any commercial or financial relationships that could be construed as a potential conflict of interest.
